# Communication interventions in adult and pediatric oncology: A scoping review and analysis of behavioral targets

**DOI:** 10.1371/journal.pone.0221536

**Published:** 2019-08-22

**Authors:** Bryan A. Sisk, Ginny L. Schulz, Jennifer W. Mack, Lauren Yaeger, James DuBois

**Affiliations:** 1 Department of Pediatrics, Division of Hematology/Oncology, Washington University School of Medicine, St. Louis, Missouri, United States of America; 2 Pediatric Oncology and Division of Population Sciences, Dana-Farber Cancer Institute, Boston, Massachusetts; and Division of Pediatric Hematology/Oncology, Boston Children’s Hospital, Boston, Massachusetts, United States of America; 3 Becker Library, Washington University School of Medicine, St. Louis, MO, United States of America; 4 Department of Medicine, Division of General Medical Sciences, Washington University School of Medicine, St. Louis, Missouri, United States of Ameica; University of Birmingham, UNITED KINGDOM

## Abstract

**Background:**

Improving communication requires that clinicians and patients change their behaviors. Interventions might be more successful if they incorporate principles from behavioral change theories. We aimed to determine which behavioral domains are targeted by communication interventions in oncology.

**Methods:**

Systematic search of literature indexed in Ovid Medline, Embase, Scopus, Cochrane Database of Systematic Reviews, Cochrane Central Register of Controlled Trials, Clinicaltrials.gov (2000–October 2018) for intervention studies targeting communication behaviors of clinicians and/or patients in oncology. Two authors extracted the following information: population, number of participants, country, number of sites, intervention target, type and context, study design. All included studies were coded based on which behavioral domains were targeted, as defined by Theoretical Domains Framework.

**Findings:**

Eighty-eight studies met inclusion criteria. Interventions varied widely in which behavioral domains were engaged. Knowledge and skills were engaged most frequently (85%, 75/88 and 73%, 64/88, respectively). Fewer than 5% of studies engaged social influences (3%, 3/88) or environmental context/resources (5%, 4/88). No studies engaged reinforcement. Overall, 7/12 behavioral domains were engaged by fewer than 30% of included studies. We identified methodological concerns in many studies. These 88 studies reported 188 different outcome measures, of which 156 measures were reported by individual studies.

**Conclusions:**

Most communication interventions target few behavioral domains. Increased engagement of behavioral domains in future studies could support communication needs in feasible, specific, and sustainable ways. This study is limited by only including interventions that directly facilitated communication interactions, which excluded stand-alone educational interventions and decision-aids. Also, we applied stringent coding criteria to allow for reproducible, consistent coding, potentially leading to underrepresentation of behavioral domains.

## Introduction

Effective communication is essential to optimize the experiences of patients with cancer. However, “effective communication” ca be defined in many ways. In 2007, a National Cancer Institute consortium defined the following six core functions of patient-centered communication in oncology: exchanging information, enabling self-management, making decisions, fostering a healing relationship, responding to emotions, and managing uncertainty.[1] Effectively fulfilling these communication functions has been associated with better mental health and lower healthcare expenditures,[2] as well as improved hope,[3] trust in the oncologist,[4] satisfaction with medical care,[5] and peace of mind.[6] However, a large body of evidence shows that clinicians often fail to fulfill many of these communication functions.[7–16]

Fulfilling all of these communication functions, however, is a difficult task. It is no surprise that many clinical teams might struggle to effectively communicate with patients and their families. Improving communication in medicine requires that clinicians and patients change their behaviors, sometimes in ways that are unfamiliar or uncomfortable. Many interventions to improve communication have been tested, but with variable success. To develop more successful communication interventions, we propose that investigators begin to view communication as a complex clinician behavior influenced by cognitive, social, economic, and cultural factors.[17, 18]. If viewed in this way, investigators can use the lens of behavioral change domains to identify novel targets for communication interventions, as we have previously argued.[19]

In psychology, models of behavioral change have sought to understand how individuals will behave in certain circumstances by evaluating multiple determinants that affect the behavior of interest. Other complex clinician behaviors, like prescribing practices and compliance with antibiotic stewardship, have been amenable to behavioral change theories.[20, 21] However, investigators have not rigorously or specifically applied these concepts to communication behaviors. We propose that investigators should further incorporate principles from the psychology of behavior change into the conceptualization and design of communication interventions.

More than 80 theories of behavioral change have been published, each with different strengths and weaknesses.[22] The Theoretical Domains Framework (TDF) was developed to consolidate multiple theories and theoretical constructs into a single framework with 14 domains. As described by Atkins, et al., TDF resulted from “a collaboration of behavioral scientists and implementation researchers who identified theories relevant to implementation and grouped constructs from these theories into domains. The collaboration aimed to provide a comprehensive, theory-informed approach to identify determinants of behavior.”[23] TDF is a theoretical framework that “provides a theoretical lens through which to view the cognitive, affective, social and environmental influences on behavior.”[23] TDF has been applied to several areas of clinical practice, including adherence to surgical best practices,[24] opioid prescription,[25] and reporting of medication errors by clinicians,[26] among many others. TDF can also serve as a lens for identifying potential levers for changing communication behaviors.

In this article, we report the results of a scoping review focused on recent communication interventions in pediatric and adult oncology, posing the question “Which domains of behavioral change are targeted by communication intervention studies in oncology?” While several previously published review articles have focused on specific modalities of communication interventions, no review has broadly evaluated the full field of communication interventions, nor has any review evaluated which behavioral domains are targeted by interventions. By identifying these behavioral domains, we aimed to highlight areas for further innovation in the development of communication interventions.

## Methods

We conducted a systematic search and scoping review following recently published Preferred Reporting Items for Systematic Reviews and Meta-Analyses (PRISMA) guidelines for scoping reviews.[27] We did not register a review protocol. For the PRSMA scoping review checklist, see S1 Checklist.

### Data sources and searches

A medical librarian (LHY) searched published literature for the concepts of ‘oncology patients’, ‘clinical communication’, ‘communication skills’, and ‘training interventions’. Due to the broad nature of search terms used to capture these concepts the search was built for specificity using major focus controlled vocabulary terms, proximity searching, and keywords in Ovid Medline 1946-, Embase 1947-, Scopus 1823-, Cochrane Database of Systematic Reviews (CDSR), Cochrane Central Register of Controlled Trials (CENTRAL), Clinicaltrials.gov 1997-, The Cumulative Index to Nursing and Allied Health Literature (CINAHL) 1937-, and PsycINFO 1800s -. Fully reproducible search strategies for each database are presented in Table 1.

**Table 1:** Full search strategies.

**Embase** Date Searched: 10/4/2018 Applied Database Supplied Limits: n/a Number of Results: 1186 Full Search Strategy: ('cancer patient'/mj OR 'advanced cancer'/mj OR 'childhood cancer'/mj OR ('paediatric oncology' OR 'pediatric oncology'):ti,ab,kw OR (cancer NEAR/8 (medicine OR patient* OR sufferer* OR advanced OR outpatient*)):ti,ab,kw OR 'oncology'/mj OR cancerology:ti,ab,kw OR (Oncolog* NEAR/5 (patient* OR fellow* OR clinical OR medical OR inpatient*)):ti,ab,kw) AND ('communication skill'/mj OR 'interpersonal communication'/mj OR 'doctor patient relation'/mj OR 'nurse patient relationship'/exp OR ((communication NEAR/2 intervention*):ti,ab,kw) OR ((communication NEAR/3 (support OR interpersonal OR skill*)):ti,ab,kw) OR ((communication* NEAR/3 (bevahior* OR behavior*)):ti,ab,kw)) AND ('training'/mj OR ((training NEAR/5 (skill OR skills OR needs OR program OR programs OR programme OR programmes OR graduate OR postgraduate OR curricula OR intervention*)):ti,ab,kw) OR ((course* NEAR/3 (training OR content OR attendance OR attenders OR multiday)):ti,ab,kw) OR 'workshops'/exp OR 'workshop'/exp OR workshop:ti,ab,kw OR workshops:ti,ab,kw OR (((intervention OR interventions) NEAR/5 (computerized OR design* OR improv* OR training*)):ti,ab,kw) OR 'teaching'/mj OR ((teaching NEAR/3 (model* OR trainees)):ti,ab,kw) OR 'education program'/exp OR ((education NEAR/3 (postgraduate OR graduate OR medical OR nurse* OR physician OR staff OR continuing)):ti,ab,kw) OR 'postgraduate education'/exp OR 'nursing education'/mj OR 'staff training'/exp OR ((preworkshop OR postworkshop) NEAR/4 encounter*) OR (((trained OR untrained) NEAR/3 (physician* OR nurse OR nurses OR staff)):ti,ab,kw) OR 'interact cancer':ti,ab,kw OR ((cai NEAR/2 (program* OR programme*)):ti,ab,kw) OR ((intervention* NEAR/8 communication*):ti,ab,kw))
**Ovid Medline** Date Searched: 10/4/2018 Applied Database Supplied Limits: Number of Results: 611 Full Search Strategy: ((paediatric oncology OR pediatric oncology).mp. OR (cancer ADJ8 (medicine OR patient* OR sufferer* OR advanced OR outpatient*)).mp. OR *MEDICAL ONCOLOGY/ OR *Oncology Service, Hospital/ OR cancerology.mp. OR (Oncolog* ADJ5 (patient* OR fellow* OR clinical OR medical OR inpatient*)).mp.) AND (*Physician-Patient Relations/ OR *Nurse-Patient Relations/ OR (communication adj2 intervention*).mp. OR (communication adj3 (support OR interpersonal OR skill*)).mp. OR (communication* adj3 (bevahior* OR behavior*)).mp.) AND (((training ADJ5 (skill OR skills OR needs OR program OR programs OR programme OR programmes OR graduate OR postgraduate OR curricula OR intervention*)).mp.) OR ((course* ADJ3 (training OR content OR attendance OR attenders OR multiday)).mp.) OR workshop.mp. OR workshops.mp. OR (((intervention OR interventions) ADJ5 (computerized OR design* OR improv* OR training*)).mp.) OR *TEACHING/ OR ((teaching ADJ3 (model* OR trainees)).mp.) OR ((education ADJ3 (postgraduate OR graduate OR medical OR nurse* OR physician OR staff OR continuing)).mp.) OR *Education, Medical, Graduate/ OR *Education, Nursing/ OR ((preworkshop OR postworkshop) ADJ4 encounter*).mp. OR (((trained OR untrained) ADJ3 (physician* OR nurse OR nurses OR staff)).mp.) OR interact cancer.mp. OR ((cai ADJ2 (program* OR programme*)).mp.) OR ((intervention* ADJ8 communication*).mp.))
**Scopus** Date Searched: 10/4/2018 Applied Database Supplied Limits: Number of Results: 1268 Full Search Strategy: ((TITLE-ABS-KEY(“paediatric oncology” OR “pediatric oncology”)) OR (TITLE-ABS-KEY(cancer W/8 (medicine OR patient* OR sufferer* OR advanced OR outpatient*))) OR (TITLE-ABS-KEY(oncology OR cancerology)) OR (TITLE-ABS-KEY(Oncolog* W/5 (patient* OR fellow* OR clinical OR medical OR inpatient*)))) AND ((TITLE-ABS-KEY(communication W/2 intervention*)) OR (TITLE-ABS- KEY(communication W/3 (support OR interpersonal OR skill*))) OR (TITLE-ABS-KEY(communication* W/3 (bevahior* OR behavior*)))) AND ((TITLE-ABS-KEY(training W/5 (skill OR skills OR needs OR program OR programs OR programme OR programmes OR graduate OR postgraduate OR curricula OR intervention*))) OR (TITLE-ABS-KEY(course* W/3 (training OR content OR attendance OR attenders OR multiday))) OR (TITLE-ABS-KEY(workshop OR workshops)) OR (TITLE-ABS-KEY((intervention OR interventions) W/5 (computerized OR design* OR improv* OR training*))) OR (TITLE-ABS-KEY(teaching W/3 (model* OR trainees))) OR (TITLE-ABS-KEY(education W/3 (postgraduate OR graduate OR medical OR nurse* OR physician OR staff OR continuing))) OR (TITLE-ABS-KEY((preworkshop OR postworkshop) W/4 encounter*)) OR (TITLE-ABS-KEY((trained OR untrained) W/3 (physician* OR nurse OR nurses OR staff))) OR (TITLE-ABS-KEY(“interact cancer”)) OR (TITLE-ABS-KEY(cai W/2 (program* OR programme*))) OR (TITLE-ABS-KEY(intervention* W/8 communication*)))
**CINAHL** Date Searched: 10/4/2018 Applied Database Supplied Limits: Number of Results: 294 Full Search Strategy: ((MM "Cancer Patients") OR (MM "Childhood Neoplasms") OR (“paediatric oncology” OR “pediatric oncology”) OR (cancer N8 (medicine OR patient* OR sufferer* OR advanced OR outpatient*)) OR (MM "Oncology") OR cancerology OR (Oncolog* N5 (patient* OR fellow* OR clinical OR medical OR inpatient*))) AND ((MM "Communication Skills") OR (MM "Physician-Patient Relations") OR (MM "Nurse-Patient Relations") OR (communication adj2 intervention*) OR (communication adj3 (support OR interpersonal OR skill*)) OR (communication* adj3 (bevahior* OR behavior*))) AAND (((training N5 (skill OR skills OR needs OR program OR programs OR programme OR programmes OR graduate OR postgraduate OR curricula OR intervention*))) OR ((course* N3 (training OR content OR attendance OR attenders OR multiday))) OR (MM "Seminars and Workshops") OR workshop OR workshops OR (((intervention OR interventions) N5 (computerized OR design* OR improv* OR training*))) OR (MM "Teaching") OR ((teaching N3 (model* OR trainees))) OR ((education N3 (postgraduate OR graduate OR medical OR nurse* OR physician OR staff OR continuing))) OR ((preworkshop OR postworkshop) N4 encounter*) OR (((trained OR untrained) N3 (physician* OR nurse OR nurses OR staff))) OR “interact cancer” OR ((cai N2 (program* OR programme*))) OR ((intervention* N8 communication*)))
**PsycInfo** Date Searched: 10/4/2018 Applied Database Supplied Limits: Number of Results: 68 Full Search Strategy: ((MM "Terminal Cancer") OR (“paediatric oncology” OR “pediatric oncology”) OR (cancer N8 (medicine OR patient* OR sufferer* OR advanced OR outpatient*)) OR (MM "Oncology") OR cancerology OR (Oncolog* N5 (patient* OR fellow* OR clinical OR medical OR inpatient*))) AND ((MM "Communication Skills") OR (MM "Interpersonal Communication") OR (communication adj2 intervention*) OR (communication adj3 (support OR interpersonal OR skill*)) OR (communication* adj3 (bevahior* OR behavior*))) AND ((MM "Training") OR ((training N5 (skill OR skills OR needs OR program OR programs OR programme OR programmes OR graduate OR postgraduate OR curricula OR intervention*))) OR ((course* N3 (training OR content OR attendance OR attenders OR multiday))) OR (MM "Seminars and Workshops") OR workshop OR workshops OR (((intervention OR interventions) N5 (computerized OR design* OR improv* OR training*))) OR (MM "Teaching") OR ((teaching N3 (model* OR trainees))) OR (MM "Postgraduate Training") OR (MM "Nursing Education") OR ((education N3 (postgraduate OR graduate OR medical OR nurse* OR physician OR staff OR continuing))) OR ((preworkshop OR postworkshop) N4 encounter*) OR (((trained OR untrained) N3 (physician* OR nurse OR nurses OR staff))) OR “interact cancer” OR ((cai N2 (program* OR programme*))) OR ((intervention* N8 communication*)))
**Cochrane Library** Date Searched: 10/4/2018 Applied Database Supplied Limits: Number of Results: CDSR: 1 CENTRAL: 257 Full Search Strategy: ((“paediatric oncology” OR “pediatric oncology”):ti,ab,kw OR (cancer NEAR/8 (medicine OR patient* OR sufferer* OR advanced OR outpatient*)):ti,ab,kw OR [mh ^"medical oncology"] OR [mh ^“Oncology Service, Hospital”] OR cancerology:ti,ab,kw OR (Oncolog* NEAR/5 (patient* OR fellow* OR clinical OR medical OR inpatient*)):ti,ab,kw) AND ([mh ^”Physician-Patient Relations”] OR [mh ^”Nurse-Patient Relations”] OR ((communication NEAR/2 intervention*):ti,ab,kw) OR (communication NEAR/3 (support OR interpersonal OR skill*)):ti,ab,kw OR (communication* NEAR/3 (bevahior* OR behavior*)):ti,ab,kw) AND ((training NEAR/5 (skill OR skills OR needs OR program OR programs OR programme OR programmes OR graduate OR postgraduate OR curricula OR intervention*)):ti,ab,kw OR (course* NEAR/3 (training OR content OR attendance OR attenders OR multiday)):ti,ab,kw OR workshop:ti,ab,kw OR workshops:ti,ab,kw OR ((intervention OR interventions) NEAR/5 (computerized OR design* OR improv* OR training*)):ti,ab,kw OR [mh ^“TEACHING”] OR (teaching NEAR/3 (model* OR trainees)):ti,ab,kw OR (education NEAR/3 (postgraduate OR graduate OR medical OR nurse* OR physician OR staff OR continuing)):ti,ab,kw OR [mh ^”Education, Medical, Graduate”] OR [mh ^”Education, Nursing”] OR ((preworkshop OR postworkshop) NEAR/4 encounter*):ti,ab,kw OR ((trained OR untrained) NEAR/3 (physician* OR nurse OR nurses OR staff)):ti,ab,kw OR “interact cancer”:ti,ab,kw OR (cai NEAR/2 (program* OR programme*)):ti,ab,kw OR (intervention* NEAR/8 communication*):ti,ab,kw) ClinicalTrials.gov Date Searched: 10/5/2018 Number of Results: 8 Full Search Strategy: "cancer patient" AND "communication skills" AND (training OR workshop) AND (clinician OR physician OR nurse)

### Study selection

This review was inclusive of research articles presenting original data on interventions to facilitate communication between clinicians and patients (or parents of pediatric patients) in oncology. Exclusion criteria included: manuscripts published in non-English language; not an intervention; not focused on communication related to cancer; not focused on actual or potential clinical encounter (i.e. cancer scenario used for training with non-oncology professionals or students); abstract or conference presentation; protocol only without results; no pre/post assessment or control comparison pertinent to communication functions or outcomes; secondary analysis of previously published intervention; published prior to year 2000; not targeting either patients or clinicians who primarily see cancer patients; study sample with fewer than 30 participants. We focused on articles published after 2000 to narrow focus to the current state of the field. We utilized the cutoff of 30 participants as an initial screen of quality, anticipating that studies with fewer than 30 participants would be pilot studies with limited external validity. If a study included clinicians and patients, we used the larger number to determine eligibility. For example, if a study included 10 clinicians, but assessed outcomes of 40 patients, we included this study in analysis. One author (BAS) screened study titles and abstracts prior to detailed review of full text. After full text review, this author excluded studies that did not meet the inclusion/exclusion criteria.

### Data synthesis and analysis

All included articles were coded based on (1) which core functions of patient-clinician communication each study addressed, and (2) which behavioral domains each intervention directly engaged, using definitions provided in Table 2. Coding definitions for communication functions were based on definitions initially described by Epstein and Street in 2007,[1] and previously modified and employed by our group in two prior publications.[28, 29] Definitions for behavioral domains were based on the refined Theoretical Domains Framework definitions published in 2012.[30] Of the 14 total domains listed in the Theoretical Domains Framework, we excluded “memory, attention, and decision process” and “optimism” after the authorship group determined that these domains were less relevant to communication. Definitions were refined after coding the first 10 articles by two reviewers (BAS and GLS). These final definitions were agreed upon by all authors. For each article, these reviewers assigned one or more codes for communication functions and behavioral domains targeted by the intervention, meaning that one article could be coded as targeting multiple communication functions and behavioral domains. Agreement was good for all categories of communication functions (mean kappa for agreement = 0.82, range 0.72 to 0.89) and behavioral domains (mean kappa for agreement = 0.87, range 0.78 to 0.93). Discrepancies were resolved by consensus between the two reviewers.

**Table 2:** Definitions of communication functions and behavioral domains.

**Communication functions**	
Fostering healing relationships	Intervention aims to support the fostering of a healing relationship. Such a relationship is based on rapport and trust, and will provide guidance, and understanding. Studies focused on the role of active listening would fall into this category. Ideally, this outcome would focus specifically on the relationship, rather than topics, which might affect a relationship.
Exchanging information	Intervention aims to improve the exchange of information about the cause, diagnosis, treatment, prognosis and psychosocial aspects of the illness. These studies may take into account information needs of the patient or family.
Responding to emotions	Intervention aims to support clinicians in recognizing and/or responding to the patient's/family's emotional states: including fear, humor, nervousness, worry, sadness, or fatalistic thinking. These interventions may aim to support clinicians in recognizing a patient's emotional state, asking the appropriate questions to understand it, communicating that understanding to the patient/family, and responding. Alternatively, these interventions could support patients/families in expressing their emotions. These interventions should specifically focus on the role of emotions in the physician/parent/patient relationship, or how one party responds to emotions within this relationship.
Managing uncertainty	Intervention aims to support patients in managing uncertainty. This is distinct from exchanging information because more information in itself can lead to more uncertainty at times. Specifically, these interventions could target the manner in which a clinician deals with uncertainty when communicating with a family, how the clinician supports a patient/family in uncertainty, or the intervention could aim to directly support a patient's/family's response to uncertainty.
Making decisions	Intervention aims to support decision-making that is based on the patient's/family's needs, values, and preferences.
Enabling patient self-management	Intervention aims to support the patient's/family's ability to solve health-related problems and to take actions to improve their health. Examples of self-management include ability to find information outside the clinical encounter, cope with treatment effects, and seek appropriate care when needed.
**Behavioral domains**	
Knowledge	Interventions that aim to improve knowledge about communication skills or communication challenges.
Skills	Interventions that aim to improve communication skills, competence, ability, or provide opportunity to practice communication skills.
Social/professional role and identity	Interventions that aim to improve communication by targeting professional identity, professional role, social identity, leadership skills, group identity, or perceived professional boundaries.
Beliefs about capabilities	Interventions that aim to modify communication-related self-confidence, self-efficacy, perceived behavioral control, self-esteem, or empowerment, often through directed or systematic feedback to clinicians.
Beliefs about consequences	Interventions that aim to modify beliefs about consequences or outcomes of communication. This might include examples of communication going poorly, rather than only focusing on communication going well.
Reinforcement	Interventions that aim to reinforce certain communication behaviors with rewards, incentives, punishments, or sanctions.
Intentions	Interventions that aim to modify the will or intentions of participants. This should be a specific aim, as opposed to providing knowledge and skills that might indirectly affect the intentions.
Goals	Interventions that aim to support the development of communication goals, such as distal or proximal goal setting, goal priority, action planning. This can be exemplified by question prompt lists. While intentions can be formed from general information about the patient (e.g. needs assessments or other surveys), goals should be particularized.
Environmental context and resources	Interventions that aim to improve communication by targeting environmental stressors, resources, barriers, facilitators, organizational culture, and person/environment interaction.
Social influences	Interventions that aim to improve communication by targeting social pressures, norms, group conformity, social support, and power.
Emotion	Interventions that aim to improve communication by targeting emotions such as anxiety, fear, stress, depression, or burnout.
Behavioral regulation	Interventions that aim to improve communication by supporting breaking of habits and self-monitoring. This might include reflective checklists.

Two authors (BAS and GLS) extracted the following information from included studies: population, whose behavior was targeted by interventions, number of study participants, type of intervention, study design, context of clinical communication, country, and number of sites. One author (BAS) subsequently extracted the following additional information: outcome measures utilized, positive and null outcomes reported, whether primary outcome was defined within article, and technology utilized by interventions. Notably, if an article performed statistical analyses on every question within a scale, we still counted the entire scale as a single outcome measure. We applied the same approach to studies that performed statistical analyses on the coding of multiple individual behaviors in recorded interactions. All data was charted independently in Excel spreadsheets by two authors (BAS and GLS).

### Role of the funding source

Financial support for this study was provided in part by grants from National Center for Advancing Translational Sciences of the National Institutes of Health and the American Society of Clinical Oncology Young Investigator’s Award. The funding agreements ensured the authors’ independence in designing the study, interpreting the data, writing, and publishing the report.

## Results

**Fig 1:**
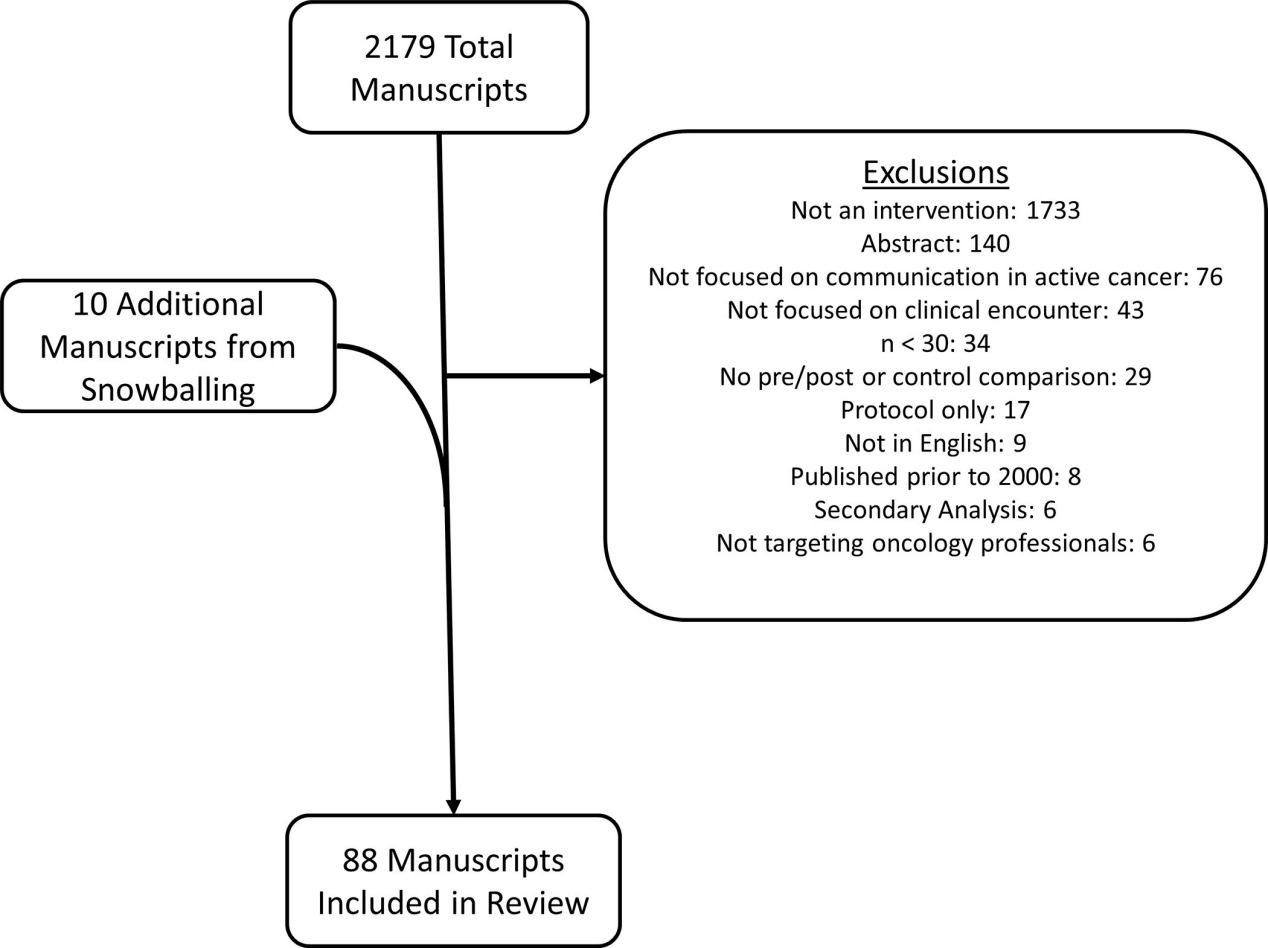
PRISMA flow diagram.

All search strategies were created and run in October 2018 finding a total of 3,692 records. Using Endnote’s automatic duplication finder 1,416 records were removed. An additional 97 duplications were removed leaving a total of 2,179 unique citations included in the project library. This search was supplemented by manual searching through reference lists and review articles, which yielded an additional 10 articles. (Fig 1) After exclusions, 88 articles remained for analysis.

### Study characteristics

Complete details of study characteristics are presented in Table 3. Eighty percent (70/88) of studies were performed in North America or Western Europe, with another 12% (11/88) in Australia/New Zealand, and 8% (7/88) in Asia. We did not identify any studies from Eastern Europe, South America, Latin America, or Africa. While 34% (30/88) of these studies were performed at a single institution, at least 22% (20/88) included 5 or more institutions. Notably, 25% (22/88) of studies reported “multiple” institutions in the methods, but did not specify the number of participating institutions. The number of clinician participants ranged from 7 to 518, with a median of 57. The number of patient participants ranged from 32 to 2314, with a median of 206. Notably, studies almost exclusively targeted adult oncology clinicians and patients (97%, 85/88). Only 3/88 studies included pediatric and adult oncology clinicians, and no studies specifically targeted communication in pediatric or adolescent oncology.

**Table 3:** Characteristics of studies.

Variable	% Yes (n)	References
**Country**		
United States + Canada	33 (29)	[31–59]
Western Europe	47 (41)	[60–100]
Europe + Australia/New Zealand	1 (1)	[101]
Australia/New Zealand	11 (10)	[102–111]
Asia	8 (7)	[112–118]
**Number of sites**		
1	34 (30)	[31–34, 37, 41, 46, 47, 49, 51, 52, 56–59, 61, 62, 64, 67, 71, 73, 84, 88, 93–95, 104, 106, 108, 118]
2	6 (5)	[40, 44, 48, 50, 114]
3	6 (5)	[55, 80, 89, 103, 110]
4	7 (6)	[53, 54, 79, 86, 115, 116]
5 to 10	11 (10)	[35, 38, 72, 78, 83, 87, 102, 105, 107, 111]
11 or more	11 (10)	[36, 39, 42, 60, 70, 74, 77, 91, 97, 109]
Multiple, but not specified	25 (22)	[43, 45, 63, 65, 66, 68, 69, 75, 76, 81, 82, 85, 90, 92, 96, 98–101, 112, 113, 117]
Number of participants: clinicians*		
30 or fewer	24 (15)	[48, 50, 65–67, 71, 72, 87, 88, 103, 105, 107, 114–116]
31 to 60	34 (21)	[35, 40, 44, 46, 49, 51, 58, 80, 82–84, 86, 93, 94, 96, 98, 99, 106, 109, 110, 113]
61 to 90	11 (7)	[74, 75, 85, 90, 92, 101, 117]
91 to 120	18 (12)	[43, 45, 59, 62, 63, 76, 77, 89, 91, 95, 97, 118]
>120	13 (8)	[31, 32, 34, 39, 57, 70, 78, 112]
Number of participants: patients*		
100 or fewer	31 (15)	[33, 41, 47, 50, 52, 53, 56, 64, 81, 85, 102, 103, 111, 115, 116]
101–200	19 (9)	[48, 54, 58, 60, 68, 79, 104, 105, 108]
201–300	25 (13)	[35, 38, 40, 42, 59, 65, 67, 71, 73, 74, 87, 95, 100]
301–400	8 (4)	[69, 72, 83, 107]
401–500	2 (1)	[88]
>500	15 (7)	[34, 36, 37, 55, 66, 101, 114]
**Population**		
Adult oncology	97 (85)	[31, 33, 35–48, 50–118]
Adult and pediatric oncology	3 (3)	[32, 34, 49]
**Whose behavior targeted by intervention**		
Attending physician	26 (23)	[36, 40, 51, 58, 60, 72, 78, 82–86, 90, 93, 94, 96, 98, 101, 106, 107, 109, 113, 114]
Fellow	5 (4)	[34, 43, 44, 46]
Nurse	20 (18)	[31, 39, 61, 68–70, 75, 77, 80, 88, 89, 91, 97, 110, 115–118]
Combined healthcare team	15 (13)	[32, 45, 49, 57, 62, 63, 66, 76, 87, 92, 95, 99, 112]
Combined patient/healthcare team	15 (13)	[35, 37, 48, 50, 56, 59, 65, 67, 71, 74, 79, 103, 105]
Patient	18 (16)	[33, 38, 41, 42, 47, 52–55, 64, 73, 81, 100, 102, 104, 108]
Patient and family	1 (1)	[111]
**Study design**		
Quasi-experimental pre/post	38 (33)	[31, 32, 34, 43–47, 49, 51, 52, 56, 57, 61–63, 68, 69, 72, 75, 76, 78, 79, 82, 83, 99, 103, 106, 109, 110, 112, 113, 118]
RCT	59 (52)	[33, 35–42, 48, 50, 53–55, 58–60, 64, 65, 67, 70, 71, 73, 77, 80, 81, 84–86, 88–98, 100–102, 104, 105, 107, 108, 111, 114–117]
Other	3 (3)	[66, 74, 87]
**Type of intervention**		
Communication skills training/educational curriculum	68 (59)	[31, 32, 34, 39, 40, 43–46, 49, 51, 53, 57, 58, 60–63, 66, 68–70, 72, 75–78, 80, 82–99, 101, 103, 106, 107, 109, 110, 112–118]
Question prompt list	3 (3)	[33, 105, 111]
Patient-directed educational intervention	6 (5)	[41, 42, 47, 81, 102]
Communication or shared decision-making coaching	4 (3)	[52, 54, 64]
Patient Needs/Symptom/Preference Assessment	8 (8)	[50, 55, 56, 65, 67, 71, 79, 108]
Multimodal combination of interventions	7 (6)	[35, 37, 38, 48, 59, 74]
Other	4 (4)	[36, 73, 100, 104]
**Context of clinical communication**		
General	50 (43)	[31, 32, 34, 40, 46–49, 57, 58, 60, 62, 66–70, 72, 74, 76, 79, 81–83, 88, 90–98, 100, 106, 107, 109, 110, 112–114, 118]
End of life/palliative care	14 (13)	[35, 43, 44, 59, 61, 64, 71, 77, 84, 105, 108, 111, 117]
Cancer treatment/decision making	12 (11)	[36–39, 41, 52, 55, 56, 85, 101, 102]
New diagnosis/prognosis	9 (8)	[33, 45, 51, 73, 80, 104, 115, 116]
Pain/symptom management	8 (7)	[42, 50, 53, 54, 75, 78, 89]
Clinical trial enrollment	7 (6)	[63, 65, 86, 87, 99, 103]

*Not all studies targeted clinicians and patients, therefore total numbers in each category are less than the total number of studies.

Fifty-nine percent (52/88) of studies employed randomized controlled trial (RCT) study designs, with most of the remainder employing quasi-experimental pre/post assessment methodology (38%, 33/88). The majority of studies (68%, 59/88) employed communication skills training/communication educational curricula. Only 7% (6/88) of studies employed multimodal interventions (e.g. communication skills training and question prompt lists utilized in the same study), with the remaining 93% (82/88) of studies employing unimodal interventions. Only 17 studies (19%) utilized technology to facilitate communication, beyond utilizing audio- and video-recordings to evaluate interventions. Most of these interventions targeted patients: video preparation for patients prior to consultation,[38, 41, 81, 102] providing patients with recordings of consultations,[73, 81] computer-assisted needs assessment, symptom monitoring, and/or question prompt sheet,[37, 50, 55, 59, 67, 71, 81] web-based decision-support intervention,[56] communication coaching via telephone,[108] and integration of interventions into the electronic medical record.[59] Some technological interventions also targeted clinicians: computer-assisted communication training for clinicians [40, 72, 74] or delivering communication skills training via teleconference.[107]

The context of communication in all included articles was mostly “general” communication without further specification within the article (50%, 43/88). The remainder were distributed among the following topics: end of life/palliative care (14%, 13/88), cancer treatment/decision making (12%, 11/88), new diagnosis/prognosis (9%, 8/87), pain/symptom management (8%, 7/88), clinical trial enrollment (7%, 6/88).

### Communication functions targeted

**Fig 2:**
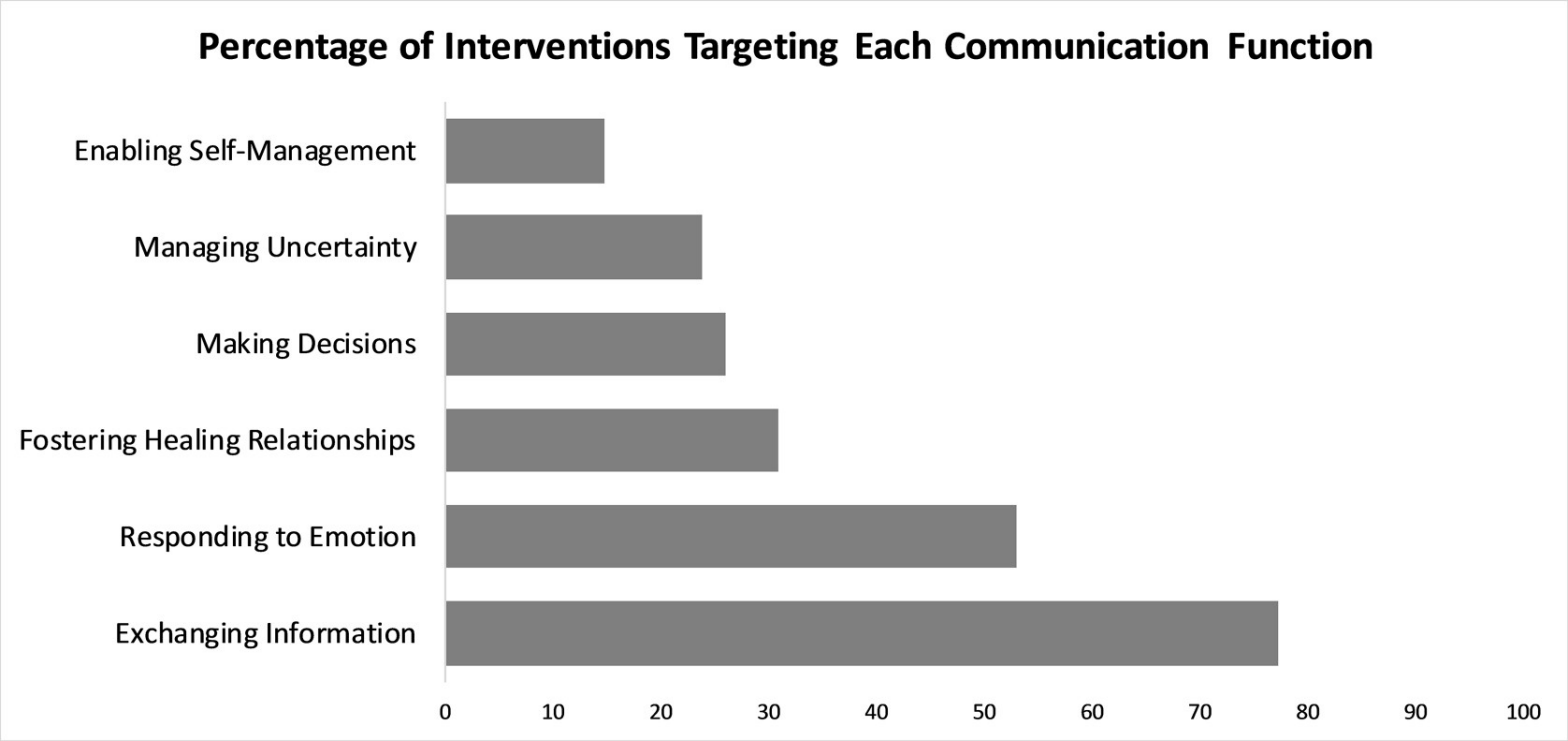
Percentage of interventions targeting each communication function. Each study was considered a single intervention; therefore, percentage represents percentage of total studies included in this review.

We found evidence that all 6 communication functions were targeted by studies included in this review. The frequency with which these studies targeted the 6 communication functions ranged from 77% (68/88) for exchanging information to 15% (13/88) for enabling self-management. (Fig 2) Notably, 8% (7/88) articles did not provide sufficient methodological information to determine which communication functions were targeted.

### Behaviors and behavioral domains

Interventions targeted the behaviors of individuals with a wide variety of roles, with 31% targeting physicians (23/88 attending physicians and 4/88 fellows), 20% (18/88) targeting nurses, 15% (13/88) targeting multiple healthcare team members, 15% (13/88) targeting members of the healthcare team and the patient, and 18% (16/88) targeting the patient.

**Fig 3:**
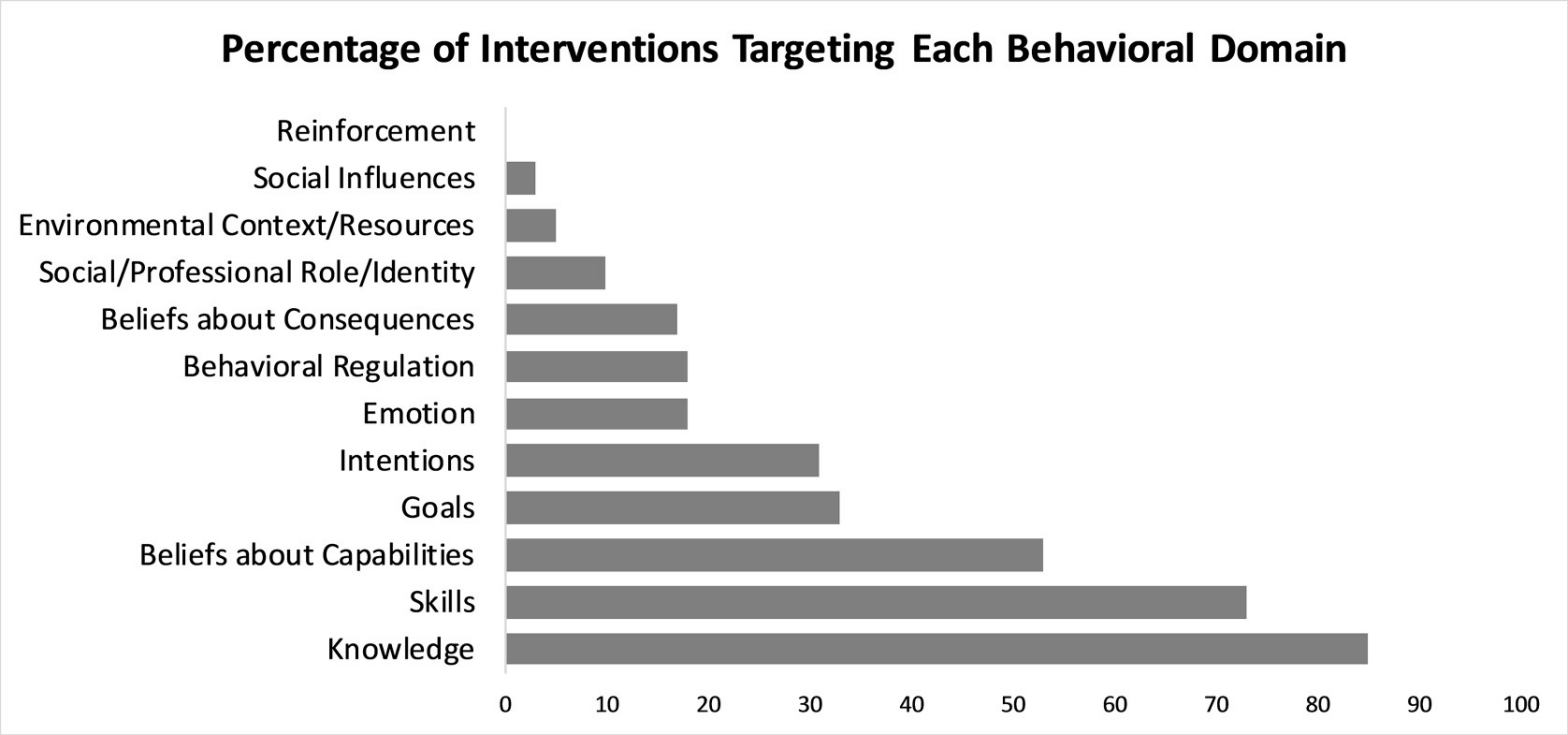
Percentage of interventions targeting each behavioral domain. Each study was considered a single intervention; therefore, percentage represents percentage of total studies included in this review.

Interventions varied widely in which behavioral domains they engaged. (Fig 3) While knowledge and skills were engaged most frequently (85%, 75/88 and 73%, 64/88, respectively), fewer than 5% of studies engaged social influences (3%, 3/88) or environmental context/resources (5%, 4/88). No studies engaged reinforcement. Overall, 7 of these 12 behavioral domains were engaged by fewer than 30% of included studies.

Complete details regarding communication functions, behavioral domains, and study outcomes for each study are available in Table 4.

**Table 4:** Outcomes and targets of individual studies.

Study	Type of intervention	Whose behavior targeted	Study design	Time point of evaluation (in addition to baseline)	Positive outcomes	Negative outcomes	Behavioral domains targeted	Communication functions targeted
Back et al. 2007[43]	Communication skills training/educational curriculum	Fellow	Quasi-experimental pre/post	Immediately post-training	Skill acquisition for “perception,” “invitation,” “knowledge,” “emotion.” Also, skill acquisition for empathic skills–“naming,” “respecting,” “supporting,” and “exploring.”	No significant skill acquisition for “summarizing” or “understanding.”	Knowledge, skills, beliefs about capabilities, behavioral regulation.	Fostering healing relationship, exchanging information, making decisions, managing uncertainty
Banerjee et al. 2017[31]	Communication skills training/educational curriculum	Nurse	Quasi-experimental pre/post	Immediately post-training	Improvement in nurse self-efficacy in the following domains: responding empathically to patients; discussing death, dying, and end-of-life goals of care; and responding to challenging family interactions. Improvement in skills related to clarifying questions, empathic communication, encouraging expressions of feelings, normalizing, praising patient efforts, and composite measure of all skills.	No significant improvement in the following skills: agenda setting (declaring agenda, inviting agenda, negotiating agenda, taking stock), checking (checking understanding, checking preference), questioning (asking open questions, restating, endorsing question asking, inviting questions), information organization (previewing, summarizing, transitioning, and reviewing next steps), acknowledging, and validating.	Knowledge, skills, beliefs about capabilities.	Fostering healing relationship, making decisions, responding to emotions
Baughcum et al. 2007[44]	Communication skills training/educational curriculum	Fellow	Quasi-experimental pre/post	Immediately post-training	Increase in fellows’ knowledge of grief/bereavement, pediatric issues, pain, and symptom management	No change in fellows’ knowledge of ethics or communication.	Knowledge and skills	Unclear/not enough information
Bernhard et al. 2012[101]	Communication skills training/educational curriculum	Attending physician	Rct	Immediately after consultation, 2 weeks post-consultation, and 4 months post-consultation	None	No improvement in decisional conflict, patient involvement in decision making, or satisfaction with doctor’s consultation skills	Knowledge and skills	Exchanging information, making decisions
Bialer et al. 2011[32]	Communication skills training/educational curriculum	Combined healthcare team	Quasi-experimental pre/post	Immediately post-training	Increase in participants’ confidence in responding to patient anger	None	Knowledge, skills, beliefs about capabilities, emotion	Fostering healing relationships, responding to emotions
Brown et al. 2010[45]	Communication skills training/educational curriculum	Combined healthcare team	Quasi-experimental pre/post	Immediately post-training	Increase in participants’ confidence in discussing prognosis	None	Knowledge, skills, beliefs about capabilities	Fostering healing relationships, exchanging information, responding to emotions, managing uncertainty
Brown et al. 2010[46]	Communication skills training/educational curriculum	Fellow	Quasi-experimental pre/post	Immediately post-training	Increase in participants’ confidence related to the following modules: breaking bad news, shared treatment decision making, responding to patient anger, discussing prognosis, discussing the transition to palliative care, discussing dnr orders. On videorecording, participants demonstrated more skill usage than prior to training.	None.	Knowledge, skills	Fostering healing relationship, exchanging information, making decisions, responding to emotion, managing uncertainty
Brown et al. 2007[103]	Communication skills training/educational curriculum	Attending physician	Quasi-experimental pre/post	Immediately post-training	Increase in number of behaviors from the shared decision-making domain (4 of 14 domains significantly improved). Also, increase in percentage of oncologists demonstrating “enactment,” describing standard treatment, non-maleficence, discussion of randomization related to bias, providing information about other trials suitable for the patient. Decrease in physician demonstrating favoring one option.	Of 58 behaviors recorded, 48 had no significant change pre/post-intervention.	Knowledge, skills, beliefs about capabilities, goals	Exchanging information, making decisions
Brown et al. 2004[102]	Patient-directed educational intervention	Patient	Rct	Audiotape analysis immediately post-intervention; satisfaction survey immediately after intervention and 2 weeks post-intervention	Increase in patients’ declaration of cost perspectives and benefit perspectives.	No change in patient preferences for information, involvement in decision making, decisional conflict, anxiety, or depression. Also, no change 31 of 33 coded communication behaviors.	Knowledge, beliefs about consequences, intentions, goals	Exchanging information, making decisions
Bruera et al. 2003[33]	Question prompt list	Patient	Rct	Immediately post-intervention	Increase in rating of “helpfulness of written material” and “written material helped to communicate with the doctor.” Also, increase in number of questions asked about diagnosis for intervention group.	No increase in ratings of “overall satisfaction with communication with the doctor,” “satisfaction with the consult,” “doctor was able to answer my questions,” or “will use similar written material in the future.” No difference in duration of consultation, number of questions asked by the patient, minutes the patient spoke, minutes the doctor spoke, number of questions on treatment, prognosis, or other issues.	Intentions, goals	Exchanging information
Butow et al. 2004[104]	Other–cancer consultation preparation package	Patient	Rct	Immediately post-intervention and 1 month post-intervention	Increase in number of questions asked by patients, which was driven by questions about prognosis.	No difference in number of clarification questions. No difference in summed active patient behaviors. No difference in number of critical information items provided by physician, physician rapport building behaviors, encouraging patient participation, consultation length, or amount of time physician and patient spent speaking.	Knowledge, intentions, goals, environmental context and resources	Exchanging information, making decisions, enabling self-management
Bylund et al. 2018[34]	Communication skills training/educational curriculum	Fellow	Quasi-experimental pre/post	1 month post-training	Increase in physician self-confidence for each of the following modules: breaking bad news, shared decision making, responding to patient anger, discussing prognosis, transition to palliative care, end of life goals of care discussions, working with interpreters, and responding to adverse events. Of 27 skills measured, significant uptake of 18 skills in in interaction with standardized patients, but only uptake of 4 skills in actual patient encounters.	No increased uptake of skills in 9 of 27 skills in interaction with standardized patients, but no uptake in 23 of 27 skills in actual patient encounters. No increase in 27 patient evaluative items after interaction with physician.	Knowledge, skills, social / professional role and identity	Exchanging information, making decisions, responding to emotion
Bylund et al. 2011[47]	Patient-directed educational intervention	Patient	Quasi-experimental pre/post	Immediately post-training	Increase in scores on patient report of communication behaviors (prcb)	None reported	Knowledge, skills, beliefs about capabilities	Exchanging information, making decisions, managing uncertainty
Canivet et al. 2014[89]	Communication skills training/educational curriculum	Nurse	Rct	Immediately post-training and 3 months post-training	Increase in nurses asking questions about emotional component of cancer pain, assessment of cognitions associated with cancer pain medication, fewer paternalistic statements about cancer pain management. Also, increase in overall assessment of psychological aspects of cancer pain medication and overall conclusions about cancer pain management decisions.	No difference in 18 of 22 cancer pain management communication strategies	Knowledge, skills, beliefs about capabilities, emotion	Exchanging information
Clayton et al. 2007[105]	Question prompt list	Combined patient / healthcare team strategy	Rct	Within 24 hours of intervention, and 3 weeks after intervention	Increased number of questions asked by patients, and total number of issues raised by patients as either question or concern. Seven of 9 topics were discussed significantly more often in intervention group. Physician endorsement associated with more questions asked.	No increase in concerns raised about specific topics, or in general. Two of 9 topics were not discussed significantly more in intervention group. No difference in anxiety scores.	Social / professional role and identity, intentions, goals	Exchanging information, enabling self-management, managing uncertainty
Cornbleet et al. 2002[100]	Other–patient held medical record	Patient	Rct	4 to 6 months post-intervention	None reported.	No difference in 10 communication outcomes.	Behavioral regulation	Exchanging information
Davison et al. 2002[55]	Patient needs / symptom / preference assessment	Patient	Rct	Immediately after intervention	Patients in intervention group preferred less active role in decision making.	None reported.	Intentions, goals	Exchanging information, making decisions, responding to emotion
Davison et al. 2014[56]	Patient needs / symptom / preference assessment	Combined patient / healthcare team strategy	Quasi-experimental pre/post	Immediately after intervention	Increase patient report of assuming more active role in decision making than previously reported. Decrease in report of decision conflict related to uncertainty, being informed, values clarity, and support.	None reported.	Knowledge	Exchanging information, making decisions
Delvaux et al. 2005[90]	Communication skills training/educational curriculum	Attending physician	Rct	Immediately after intervention and 5 months post-intervention	Improvement in 2 of 16 communication skills in simulated interviews, and 11/16 communication skills in actual patient interviews.	No difference in physicians’ utterances to patients, relatives, or combination in 3-person interviews.	Knowledge, skills, beliefs about capabilities	Exchanging information, responding to emotion
Delvaux et al. 2004[91]	Communication skills training/educational curriculum	Nurse	Rct	Immediately after intervention, 3 months post-intervention, and 6 months post-intervention	Increase in sdaq (measure of psychosocial aspects of cancer) total mean score, and the following subscale scores: attitudes toward oneself, attitudes toward cancer and death, and occupational attitudes. Decrease in stress related to inadequate preparation, caring, and overall stress.	No difference in personal growth or professional relationships. No difference in stress related to lack of support, professional conflicts, death and dying, or workload.	Knowledge, skills, emotion	Responding to emotion, managing uncertainty
Detmar et al. 2002[71]	Patient needs / symptom / preference assessment	Combined patient / healthcare team strategy	Rct	Longitudinal, crossover study with minimum of 10 consecutive patients enrolled and interviews recorded and coded	Increase in mean composite communication score. More frequent discussion of social functioning, fatigue, and dyspnea.	No increase in frequency of discussion of the following domains: physical functioning, role functioning, emotional functioning, cognitive functioning, pain, insomnia, nausea, appetite loss, and constipation or diarrhea.	Intentions, goals	Exchanging information, responding to emotion
Durey et al. 2017[106]	Communication skills training/educational curriculum	Attending physician	Quasi-experimental pre/post	Immediately after intervention and 2 months post-intervention	Increase in cultural safety confidence scores for relationships, communication, and awareness immediately after intervention and 2 months after intervention. Also, increased confidence in applying culturally safe practices in 9 of 14 items.	No increased confidence in applying culturally safe practices in 5 of 14 items.	Knowledge, skills, social / professional role and identity, beliefs about capabilities, beliefs about consequences	Fostering healing relationship, exchanging information, making decisions
Eggly et al. 2017[48]	Multimodal–question prompt list and patient coaching	Combined patient / healthcare team strategy	Rct	Immediately after intervention	Intervention did not prolong interaction length. Qpl-only format increased active participation.	No difference in talk time ratios, patient perception of role in treatment decisions, or trust in physician. Intervention (qpl + coaching) arm rated as less patient-centered than control arm.	Intentions, goals	Exchanging information, enabling self-management
Epstein et al. 2017[35]	Multimodal–question prompt list, physician communication training, and patient coaching	Combined patient / healthcare team strategy	Rct	Immediately after intervention	Improvement in composite communication score, and improved engagement of patient.	No difference in quality of life measures, response to emotions, prognostic or treatment information provision.	Knowledge, skills, beliefs about capabilities, beliefs about consequences, intentions, goals, behavioral regulation	Fostering healing relationship, exchanging information, making decisions, responding to emotion, managing uncertainty
Fallowfield et al. 2012[99]	Communication skills training/educational curriculum	Combined healthcare team	Quasi-experimental pre/post	Immediately after intervention	Improvement in presence of the following information in audiotaped conversations: symptoms / palliative care, prognosis, aims of trial, medical benefit.	No difference in the presence of the following information in audiotaped conversations: voluntary nature, unknown side effects, extra effort, and right to withdraw.	Knowledge, skills, goals	Exchanging information, making decisions, managing uncertainty
Fallowfield et al. 2002[60]	Communication skills training/educational curriculum	Attending physician	Rct	3 months after intervention	Greater number of focused questions, expressions of empathy, and appropriate responses to patients’ cues in “communication skills training” group. Also, fewer leading questions.	No difference in odds of summarizing information, interruptions, checking understanding.	Knowledge, skills, beliefs about capabilities, beliefs about consequences, intentions, goals, emotion	Unclear/not enough information
Finset et al. 2003[78]	Communication skills training/educational curriculum	Attending physician	Quasi-experimental pre/post	Immediately after intervention and 2 to 6 years after intervention	Significant long term increase in self-reported skills in communicating with severely ill patients, and improvement in self-reported ability to cope with emotional factors for female physicians.	No association of course completion with knowledge of psychological factors.	Knowledge, skills, beliefs about capabilities, emotion	Exchanging information, responding to emotion, managing uncertainty
Fleissig et al. 2001[65]	Patient needs / symptom / preference assessment	Combined patient / healthcare team strategy	Rct	Immediately after intervention	Consultations not longer in intervention vs control group.	No difference in satisfaction with consultation after intervention	None clearly targeted.	Exchanging information, making decisions
Fujimori et al. 2003[113]	Communication skills training/educational curriculum	Attending physician	Quasi-experimental pre/post	Immediately after intervention and 3 months after intervention	21 items on self-rating confidence scale for communication all significantly improved after intervention.	No difference in participants’ psychological distress, depersonalization, or personal accomplishment after intervention. Increase in emotional exhaustion.	Knowledge, skills, beliefs about capabilities, beliefs about consequences	Fostering healing relationship, exchanging information, responding to emotion
Fujimori et al. 2014[114]	Communication skills training/educational curriculum	Attending physician	Rct	Immediately after intervention	Improvement in performance scales for the following skills: setting up supportive environment, considering how to deliver bad news, and providing reassurance and addressing patient’s emotions with empathic responses. Decreased patient report of depression at follow-up.	No difference in discussing additional information. No difference in patient report of anxiety at follow-up.	Knowledge, skills, beliefs about capabilities	Fostering healing relationship, exchanging information, responding to emotion
Fukui et al. 2008[115]	Communication skills training/educational curriculum	Nurse	Rct	1 week, 1 month, and 3 months after intervention	Decrease in patient report of depression and total distress.	No difference in patient report of anxiety.	Knowledge, skills, beliefs about capabilities, beliefs about consequences, behavioral regulation	Fostering healing relationship, exchanging information, responding to emotion
Fukui et al. 2009[116]	Communication skills training/educational curriculum	Nurse	Rct	1 week, 1 month, and 3 months after intervention	Improvement in nurse’s ability to detect patient’s distress after intervention.	No difference in nurse’s ability to detect patient’s distress in mixed-effects models comparing control vs. Experimental groups.	Knowledge, skills, beliefs about capabilities, beliefs about consequences, behavioral regulation	Fostering healing relationship, exchanging information, responding to emotion
Gibon et al. 2013[92]	Communication skills training/educational curriculum	Combined healthcare team	Rct	Immediately after intervention	Increase in frequency of the following utterances: directive questions, checking questions, other types of questions, total “assessment” utterances, empathy, negotiation, and emotional words.	No difference in frequency of the following utterances: open questions, open directive questions, leading questions, acknowledgment, reassurance, total “support” utterances, procedural information, other types of information, total “information” utterances, medical words, social words, total “contents” utterances.	Knowledge, skills, social / professional role and identity, beliefs about capabilities	Exchanging information, enabling self-management, responding to emotion
Girgis et al. 2009[107]	Communication skills training/educational curriculum	Attending physician	Rct	Immediately after intervention, 1 week and 3 months after intervention	Small difference in anxiety change from baseline at 1 week, but not 3 months.	No significant difference in patients’ emotional functioning, depression, or perceived needs.	Knowledge, skills, beliefs about capabilities	Fostering healing relationship, responding to emotion
Goelz et al. 2011[84]	Communication skills training/educational curriculum	Attending physician	Rct	Immediately after intervention	Improvement in skills specific to palliative care, global communication skills, and involvement of significant others.	None reported.	Knowledge, skills, goals	Fostering healing relationship, exchanging information, responding to emotion
Griffiths et al. 2015[61]	Communication skills training/educational curriculum	Nurse	Quasi-experimental pre/post	Immediately after intervention and 2 months after intervention	Nurses more likely to report that asking about concerns and emotions benefits patients, they will not get too close to their patients, their work will not become unmanageable, exploring concerns is helpful and will not distress patients, and the nurse will not become overwhelmed by the patients emotions. Improved confidence in communication on 16 items in measure.	12 of 19 items on perceptions of outcomes measure were not different pre/post-intervention. Motivation and perceived usefulness of intervention not significantly changed after intervention.	Knowledge, skills, beliefs about capabilities	Fostering healing relationship, responding to emotion
Guadagnoli et al. 2000[36]	Other–engaging institutional medical opinion leaders and providing performance feedback	Attending physician	Rct	Immediately after intervention	None reported	No difference in discussions of surgical options between two interventions	Social / professional role and identity, intentions, goals	Exchanging information, making decisions
Härter et al. 2015[85]	Communication skills training/educational curriculum	Attending physician	Rct	Immediately after intervention and 3 months after intervention	Improved physician confidence in shared decision making. Patients in intervention group reported lower anxiety and depression scales.	No difference in patient report of confidence in decision or satisfaction with decision.	Knowledge, skills, beliefs about capabilities, behavioral regulatoin	Making decisions
Henoch et al. 2013[77]	Communication skills training/educational curriculum	Nurse	Rct	Immediately after intervention and 5 to 6 months after intervention	Increase in confidence in communication.	No change in attitudes toward care for the dying.	Knowledge	Fostering healing relationship, responding to emotion
Heyn et al. 2012[79]	Patient needs assessment	Combined patient / healthcare team strategy	Other—non-randomized control trial	Immediately after intervention	Increase in emotional cues and concerns voiced by patients in intervention group.	Increase in length of consultations by 4 minutes on average.	Intentions, goals, behavioral regulation	Exchanging information, enabling self-management, responding to emotion, managing uncertainty
Hietanen et al. 2007[87]	Communication skills training/educational curriculum	Combined healthcare team	Other—case-controlled intervention study	Immediately after intervention	Increased patient perception of sufficient time given for decision making, and that physician offered therapeutic treatments outside of trial enrollment. Patients in intervention group better understood study aims of potential clinical trial.	No difference in perception of having received enough information to make a decision, making decisions independently, or expectations of toxicity severity.	Knowledge, skills, beliefs about capabilities, beliefs about consequences, behavioral regulation	Exchanging information
Hulsman et al. 2002[72]	Communication skills training/educational curriculum	Attending physician	Quasi-experimental pre/post	4 weeks and 8 weeks after intervention	Increased ratings of physicians’ quality of performance in behavioral assessment.	No difference in assessment of physicians behaviors or patient satisfaction.	Knowledge, consequences	Fostering healing relationship, exchanging information, responding to emotion, managing uncertainty
Jenkins et al. 2002[62]	Communication skills training/educational curriculum	Combined healthcare team	Rct	3 months after intervention	Improved physician attitudes towards psychosocial issues. On recorded videotape analysis, increased use of empathic expressions, open questions, appropriate responses to patient cues, and psychosocial probing.	None reported.	Knowledge, skills, beliefs about consequences, beliefs about capabilities, intentions, emotion	Unclear/not enough information
Jenkins et al. 2005[63]	Communication skills training/educational curriculum	Combined healthcare team	Quasi-experimental pre/post	Immediately after intervention	Improved scores for the following behaviors: research nurse or doctor referred to, randomization explained, patient’s understanding of randomization checked, standard treatments discussed, treatments explained, side effects discussed, patients encouraged to discuss options with family.	Decrease in use of analogy to describe randomization. No difference in scores for the following behaviors: purpose of interview defined, study defined as research, voluntary participation explained, withdrawal from study explained, uncertainty about treatment expressed, participant summarized discussion, patients encouraged to ask questions and read information sheet, or use of 6 specifically recommended phrases.	Knowledge	Exchanging information, managing uncertainty
Johnson et al. 2013[66]	Communication skills training/educational curriculum	Combined healthcare team	Other—non-randomized control study	Immediately after intervention	None reported.	No difference in the consultation and relational empathy (are) measure completed by patients.	Knowledge, skills, beliefs about capabilities	Unclear/not enough information
Jones et al. 2011[64]	Communication or shared decision-making coaching	Patient	Rct	8 weeks after intervention.	Increased report of discussions with professionals or family and friends about the future	Happiness with communication was unchanged or worse, and satisfaction with services decreased.	Emotion	Making decisions, responding to emotion
Kruijver et al. 2001[80]	Communication skills training/educational curriculum	Nurse	Rct	Immediately after intervention	Increased verbal instrumental communication behaviors in the following categories: psychosocial / feelings, psycho-social items / feelings, total open questions, medical / therapeutic items, and fewer total closed questions.	No change in 12 of 17 instrumental communication behaviors. No differences in 14 of 14 affective communication behaviors.	Knowledge, skills, emotion, behavioral regulation	Fostering healing relationship, exchanging information, responding to emotion
Langewitz et al. 2010[75]	Communication skills training/educational curriculum	Nurse	Quasi-experimental pre/post	6 months after intervention, but immediately after booster session	Increase in empathic responses, professional reassurance, and optimistic utterances. Decrease in amount of medical or therapeutic information mentioned by nurses, and decrease in counselling about medical or therapeutic issues. Increased attention to psychosocial issues. Increased length of uninterrupted speech.	No change in 53 of 63 other communication behaviors that were coded.	Knowledge, skills	Fostering healing relationship, Enabling self-management, responding to emotion
Lenzi et al. 2011[82]	Communication skills training/educational curriculum	Attending physician	Quasi-experimental pre/post	Immediately after intervention	Increase in self-efficacy, knowledge of communication skills, favorable changes in attitudes towards disclosure of medical information and assessing patients’ concerns and fears.	No difference in “got right to the point and delivered news immediately,” challenging a patient’s denial about incurable nature of cancer, downplaying gravity of a patient’s condition in order not to destroy hope, or emphasizing the high chances of controlling pain to foster hope in a dying patient.	Knowledge, skills, beliefs about capabilities, emotion	Fostering healing relationship, exchanging information, responding to emotion
Liénard et al. 2008[93]	Communication skills training/educational curriculum	Attending physician	Rct	Immediately after intervention	None reported	No difference in report of patients’ or relatives’ anxiety.	Knowledge, skills, beliefs about capabilities	Exchanging information, responding to emotion, managing uncertainty
Liénard et al. 2006[94]	Communication skills training/educational curriculum	Attending physician	Rct	Immediately after intervention	None reported	No difference in report of patients’ anxiety.	Knowledge, skills, beliefs about capabilities	Exchanging information, responding to emotion, managing uncertainty
Liu et al. 2007[118]	Communication skills training/educational curriculum	Nurse	Quasi-experimental pre/post	1 month and 6 months after intervention	Improvement in scales for basic communication skills, self-efficacy, outcome expectancy beliefs, and perceived support in the training group.	None reported.	Knowledge, skills, social / professional role and identity, beliefs about capabilities, beliefs about consequences, environmental context and resources, social influences	Unclear/not enough information
Lubrano di Ciccone et al. 2010[49]	Communication skills training/educational curriculum	Combined healthcare team	Quasi-experimental pre/post	Immediately after intervention	Increase in participants’ confidence in conducting an interview via interpreters.	None reported.	Knowledge, skills, goals, environmental context and resources	Fostering healing relationship, exchanging information
Merckaert et al. 2015[95]	Communication skills training/educational curriculum	Combined healthcare team	Rct	Multiple time points throughout intervention	Increased clinicians assessment skills, supportive skills and provided more setting information. Patients interacting with members of the trained teams asked more open questions, expressed more emotional words, and exhibited a higher satisfaction level regarding nurses’ interventions.	No change in information utterances, or contents of clinician utterances. No change in patients’ use of medical words, radiotherapy words, or social words.	Knowledge, skills, social / professional role and identity, beliefs about capabilities	Exchanging information, enabling self-management, responding to emotion
Merckaert et al. 2005[96]	Communication skills training/educational curriculum	Attending physician	Rct	Immediately after intervention	None reported.	No difference in physicians’ ability to assess patients’ distress.	Knowledge, skills	Exchanging information, responding to emotion, managing uncertainty
Meropol et al. 2013[37]	Multimodal—assessment of patient values, goals, and communication preferences; patient communication skills training; and a preconsultation physician summary report	Combined patient/healthcare team strategy	Rct	Immediately after intervention	Patient communication skill training led to increase in patients reporting that treatment decisions were easier to reach, that they were satisfied with these decisions. Patients in intervention arms also reported higher levels of satisfaction with physician communication format and discussion regarding support services and quality of life concerns.	Patient communication skill training did not increase patient report of satisfaction with discussion about diagnosis / prognosis, overall satisfaction. Also, no effect of physician summary report on outcomes.	Knowledge, intentions, goals	Exchanging information, making decisions
Meystre et al. 2013[76]	Communication skills training/educational curriculum	Combined healthcare team	Quasi-experimental pre/post	Immediately after intervention	None reported.	No improvement in working alliance as a result of intervention.	Knowledge, skills	Fostering healing relationship, making decisions, responding to emotion
Mishel et al. 2009[38]	Communication or shared decision-making coaching	Patient	Rct	Immediately after intervention	Increase in patient report of uncertainty management (cancer knowledge, problem-solving, and patient-clinician communication, driven), medical communication competence, umber and helpfulness of resources for information, and decisional regret.	No difference in how much the patient tells nurses, how much the nurses tell the patient, mood disturbances, or quality of life.	Knowledge, skills, intentions, goals	Exchanging information, making decisions, managing uncertainty
Morasso et al. 2015[83]	Communication skills training/educational curriculum	Attending physician	Quasi-experimental pre/post	1 to 2 months after intervention	Decrease in state-anxiety levels in intervention group.	None reported.	Knowledge, skills, beliefs about capabilities, emotion	Exchanging information, responding to emotion
Morita et al. 2014[117]	Communication skills training/educational curriculum	Nurse	Rct	2 to 4 months after intervention	Increase in nurse-reported confidence in caring for terminally ill and nurse perceived value of patients inner power.	No difference in nurses’ self-reported practice score, willingness to help, positive appraisal, helplessness, nurse-perceived value of being, or burnout, emotional exhaustion, depersonalization, meaning of life, or knowledge scales.	Knowledge, skills	Responding to emotion
Ong et al. 2000[73]	Other—patients provided with audiorecording of initial consultation	Patient	Rct	1 week and 3 months after intervention	Increase in patient satisfaction with consultation and recall of information.	No difference in quality of life.	None directly targeted	Exchanging information
Paladino et al. 2019[59]	Multimodal–question prompt list and communication skills training	Combined patient / healthcare team strategy	Rct	Every 2 months for 2 years or until death	Decreased anxiety symptoms reported in intervention group at 14 weeks and 24 weeks post-intervention.	No difference in median number of goals met by patients, peace scale, human connection scale, or depressive symptoms.	Knowledge, skills, beliefs about capabilities, intentions, goals, environmental context and resources	Fostering healing relationship, exchanging information, making decisions, responding to emotion
Parker et al. 2013[39]	Communication skills training/educational curriculum	Nurse	Rct	2 months after intervention	Increase in nurse report of discussing complementary and alternative medicine with patients.	No difference in patient report of discussing complementary and alternative medicine with nurses.	Knowledge, intentions, goals	Exchanging information
Post et al. 2013[50]	Multimodal—patient needs / symptom / preference assessment and patient-directed communication training	Combined patient / healthcare team strategy	Rct	Weekly during intervention and immediately after intervention	Lower average pain severity over time in intervention group.	No difference in mean pain interference scores, mean depression, or fatigue scores.	Knowledge, skills, behavioral regulation	Exchanging information, enabling self-management, managing uncertainty
Quinn et al. 2011[51]	Communication skills training/educational curriculum	Attending physician	Quasi-experimental pre/post	Immediately after intervention	Higher proportion of participants reporting comfort with cultural communication skills, but no statistical tests performed.	None reported	Knowledge	Unclear/not enough information
Rask et al. 2009[88]	Communication skills training/educational curriculum	Nurse	Rct	1 week and 3 months after intervention	None reported.	No differences in scores on measures related to communication or work-related stress.	Knowledge, skills, beliefs about capabilities	Exchanging information, responding to emotion
Razavi et al. 2002[97]	Communication skills training/educational curriculum	Nurse	Rct	Immediately after intervention and 3 months after intervention	Increased use of emotional words by trained nurses.	None reported.	Knowledge, skills	Fostering healing relationship, responding to emotion
Razavi et al. 2003[98]	Communication skills training/educational curriculum	Attending physician	Rct	Immediately after intervention	In simulated interviews, increase in use of open and open directive questions, and utterances alerting patients to reality. Also, decrease in premature reassurance. In actual patient interviews, increase in acknowledgments, empathic statements, educated guesses, and negotiations.	In simulated interviews, no difference in 11 of 22 communication skills evaluated. In actual patient interviews, no difference in 18 of 22 communication skills evaluated.	Knowledge, skills, beliefs about capabilities	Exchanging information, responding to emotion, managing uncertainty
Sargeant et al. 2011[57]	Communication skills training/educational curriculum	Combined healthcare team	Quasi-experimental pre/post	Immediately after intervention and unclear additional follow-up	Improvement in self-reported communication skills after workshops.	None reported.	Knowledge, skills, beliefs about capabilities	Exchanging information
Schofield et al. 2013[108]	Patient needs / symptom / preference assessment	Patient	Rct	Immediately after intervention	None reported.	No difference in unmet needs, psychological morbidity and distress, or healthcare-related quality of life.	Knowledge, intentions, goals, emotion	Fostering healing relationship, exchanging information, enabling self-management, responding to emotion
Sheppard et al. 2013[52]	Communication or shared decision-making coaching	Patient	Quasi-experimental pre/post	Within 3 months after intervention	Increased self-efficacy in communicating with clinicians and making treatment decisions (not statistically tested). Increased participant rating of involvement in their care.	“no other factors were associated with pics scores.”	Knowledge, skills, beliefs about capabilities, intentions, social influences	Exchanging information, making decisions, enabling self-management
Smith et al. 2010[53]	Communication skills training/educational curriculum	Patient	Rct	Immediately after intervention, and 4 weeks and 12 weeks post-baseline assessment	Decrease in patient report of pain barriers.	No difference in patient report of pmi index, pain relief, quality of life–mental component, quality of life–physical component, distress, or satisfaction with care.	Knowledge, skills, behavioral regulation	Exchanging information, enabling self-management
Stewart et al. 2007[58]	Communication skills training/educational curriculum	Attending physician	Rct	Immediately after intervention	Subanalysis for family physicians had increased communication scores.	No difference in patient centeredness of the visit, satisfaction, psychological distress, or patient feeling better.	Knowledge, skills, beliefs about capabilities, beliefs about consequences, intentions	Unclear/not enough information
Street et al. 2010[54]	Communication or shared decision-making coaching	Patient	Rct	Immediately after intervention	Increased discussion of pain concerns and increased pain-specific participation	No increase in total patient participation.	Knowledge, skills, beliefs about capabilities, intentions, goals, behavioral regulation	Exchanging information, enabling self-management
Sutherland et al. 2007[109]	Communication skills training/educational curriculum	Attending physician	Quasi-experimental pre/post	Immediately after intervention	Increased physician confidence in using techniques to deliver bad news (10 of 10 techniques evaluated). Increase in self-report of using 3 of 8 strategies to deliver bad news.	No increase in self-report of using 5 of 8 strategies to deliver bad news.	Knowledge, skills, beliefs about capabilities	Exchanging information
Tang et al. 2014[112]	Communication skills training/educational curriculum	Combined healthcare team	Quasi-experimental pre/post	Immediately after intervention	Increase in healthcare clinicians’ truth-telling preference, as well as increases in the following subscores: method of disclosure, emotional support, additional information, and setting.	None reported.	Knowledge, skills, beliefs about capabilities	Exchanging information
Tulsky et al. 2011[40]	Communication skills training/educational curriculum	Attending physician	Rct	Immediately after intervention	Increased in physician’s use of empathic statements and increase in likelihood of responding to negative emotions empathically. Patients of intervention physicians reported greater trust in the physician.	No difference in perceived empathy, therapeutic alliance scale, perceived knowledge of the patient, perceived belief that the physician cared about the patient, or perceived belief that the physician understood the patient as a whole person.	Knowledge, beliefs about capabilities, intentions, goals, behavioral regulation	Fostering healing relationship, exchanging information, responding to emotion
Turner et al. 2009[110]	Communication skills training/educational curriculum	Nurse	Quasi-experimental pre/post	Immediately after intervention	Improvement in the following subscores for somatic subscale of ghq psychological morbidity test. Increase in item about “taking an active role in caring for myself emotionally and spiritually.” Increase in 4 of 5 confidence measures related to emotional support for patients.	No difference in maslach burnout inventory or overall ghq psychological morbidity scores. No difference in perception of stress at work or outside of work (4 of 4 items), 4 of 5 items related to attitudes, or 1 of 5 items related to confidence.	Knowledge, skills, social/professional role and identity, beliefs about capabilities	Fostering healing relationship, responding to emotion, managing uncertainty
van Bruinessen et al. 2016[81]	Patient-directed educational intervention	Patient	Rct	Immediately after intervention and 3 months after intervention	None reported.	No significant improvement in communication self-efficacy resulting from the intervention.	Knowledge, skills, intentions, goals, emotion, behavioral regulation	Exchanging information, responding to emotion
van Weert et al. 2011[74]	Multimodal—communication skills training with web-enabled video feedback and a question prompt sheet	Combined patient / healthcare team strategy	Other—randomized pre-test/post-test control group study	Immediately after intervention	Increase in discussing realistic expectations. Increase in overall information and recommendation communication behaviors. Within information behaviors, increases in 7 of 14 evaluated behaviors. Within recommendation behaviors, increase in 4 of 11 evaluated behaviors.	No difference in tailored communication, affective communication, interpersonal communication, treatmet-related information, or coping information. Decrease in rehabilitation information.	Knowledge, skills, beliefs about capabilities, intentions, goals	Exchanging information, responding to emotion
Velikova et al. 2004[67]	Patient needs assessment	Combined patient / healthcare team strategy	Rct	Immediately after intervention	Increase in patient quality of life measurement, but significance lost when time incorporated into mixed-effects model.	None reported.	Intentions, goals	Exchanging information, responding to emotion
Walczak et al. 2017[111]	Question prompt list	Patient and family	Rct	Immediately after intervention and 1 month after intervention	Increase in patient for discussion of prognosis, end-of-life care, future care options and general issues not targeted by the intervention. Increased patient self-efficacy in knowing what questions to ask their doctor.	No difference in asking questions about these issues or overall question asking, patients’ health-related quality-of-life, or the likelihood that health information or shared decision-making preferences were met.	Social / professional role and identity, beliefs about capabilities, intentions, goals, behavioral regulation	Exchanging information, making decisions
Walker et al. 2005[41]	Patient-directed educational intervention	Patient	Rct	1 to 2 weeks after intervention	In exploratory subgroup analyses, minority patients in intervention group were more satisfied with the overall clinic appointment. Unmarried patients in intervention group had lower distress. Patients with history of mental health treatment in intervention group reported higher quality of life.	No overall difference in outcomes.	Knowledge, intentions, goals, social influences, emotion	Exchanging information
Wilkie et al. 2010[42]	Patient-directed educational intervention	Patient	Rct	Immediately after intervention	Small increase in providing unsolicited sensory pain information, and mentioning it before their clinicians asked for it. Increase in mean number of pain parameters discussed.	No differences in scores for analgesic adequacy, all pain indices except one, anxiety, depression, or catastrophizing coping.	Knowledge, skills, beliefs about capabilities, beliefs about consequences, intentions, goals, behavioral regulation	Exchanging information, enabling self-management
Wilkinson et al. 2002[69]	Communication skills training/educational curriculum	Nurse	Quasi-experimental pre/post	Immediately after intervention	Improvement in 9 of 9 areas of communication assessment: introduction, admission, diagnosis, present illness, previous illness, physical, social, psychological, closure.	None reported.	Knowledge, skills, beliefs about capabilities, emotion	Fostering healing relationship, exchanging information, responding to emotion
Wilkinson et al. 2003[68]	Communication skills training/educational curriculum	Nurse	Quasi-experimental pre/post	6 weeks after intervention	Improvement in 8 of 9 areas of communication assessment: introduction, admission, diagnosis, present illness, previous illness, social, psychological, closure. Improved confidence reported in 44 of 44 items.	No change in physical assessment of the patient.	Knowledge, skills, beliefs about capabilities, beliefs about consequences, emotion	Exchanging information, responding to emotion
Wilkinson et al. 2008[70]	Communication skills training/educational curriculum	Nurse	Rct	12 weeks after intervention	Increase in nurses’ communication scores, and increase in nurses’ report of confidence in communication skills.	None reported	Knowledge, skills, beliefs about capabilities, beliefs about consequences	Exchanging information, responding to emotion, managing uncertainty
Wuensch et al. 2017[86]	Communication skills training/educational curriculum	Attending physician	Rct	2 weeks after intervention	Increase in communication score on all items, for the subgroup of content-specific items, and for the global rating of communication competence. Communication confidence improved in 9 of 10 domains.	No difference in subgroup of general communication skills. No difference in confidence in 1 off 10 domains: respect of information needs.	Knowledge, skills, goals	Exchanging information

### Characteristics of study outcomes

In total, these 88 studies reported on 188 different outcome measures. Of these 188 outcome measures, 156 measures were only used by individual studies, 14 were used by 2 studies, 9 were used by 3 studies. Four outcome measures were used by more than 5 studies, including Hospital Anxiety and Depression Scale (used in 13 studies), State-Trait Anxiety scale (used in 11 studies), EORTC Quality of Life questionnaire (used in 8 studies), and Cancer Research Campaign Workshop Evaluation Manual for coding strategies (used in 6 studies). For most studies, outcomes were assessed at a single time point immediately after the intervention (61%, 54/88). The remaining thirty-nine percent (34/88) of studies evaluated outcomes at least 1 week after the intervention (ranging from 1 week to 6 years post-intervention). Twenty-six percent (23/88) of studies evaluated outcomes at multiple time points beyond the baseline assessment– 19% (17/88) evaluated outcomes at 2 time points beyond baseline, and 7% (6/88) evaluated at 3 time points beyond baseline.

Eleven studies (13%) reported all null outcomes. Of these 11 studies, 6 employed communication skills training interventions,[66, 88, 93, 94, 96, 101] one provided patients with their medical records,[100] one engaged thought leaders at institutions to institute changes in communication behaviors of surgeons,[36] one utilized patient needs assessments prior to clinic encounters,[108] one employed a patient-directed educational intervention,[81] and one employed a multimodal intervention with communication skills training, value elicitation, and a clinical question guide.[59] The remaining 77 studies (87%) reported at least 1 positive outcome, and 70 studies (79%) reported at least 1 null outcome. However, only 42 articles (48%) identified a primary outcome of the study. Of 52 randomized-controlled trials, only 17 (33%) explicitly identified a single primary outcome of the study. Furthermore, several studies performed hypothesis testing on individual questions from measures or individual skills that were observed, often without alpha-correction for multiple comparisons. This resulted in as many as 42 separate hypothesis tests in a single study, of which some were statistically significant.[61]

## Discussion

This extensive scoping review has highlighted two generalizable lessons for the broader field of communication research in medicine. First, there is a need for further innovation in the development of interventions. For example, 7 of 12 behavioral domains were infrequently targeted by studies included in this review. “Environmental context/resources” and “social influences” were targeted by 3% of studies, and “reinforcement” was not targeted by any studies. These untapped domains represent additional behavioral levers that future interventions could target. For example, an intervention that engaged administrators in modifying performance evaluation based on communication outcomes (though practically challenging) could strongly target “reinforcement.” Open reporting of patients’ evaluations of communication might target “social and professional role/identity.” Utilizing cultural liaisons to facilitate communication with minority patients might engage the “social” domain of behavioral change. By considering these behavioral domains when developing interventions, investigators stand a better chance of supporting durable changes in communication behavior.

The narrow behavioral focus reflects the predominant utilization of unimodal interventions, primarily communication skills training sessions. While education is important, it is often insufficient to lead to persistent behavioral change. The need for additional levers of change beyond education is the conceptual basis for all behavioral change models, quality improvement scholarship, and the field of dissemination and implementation science.[30, 119] Even motivated clinicians will falter if institutional norms and practices create barriers to effective communication, such as clinic scheduling practices, large patient volumes, and clinicians’ time constraints.[120–124] To overcome these barriers, future interventions should use multimodal approaches to target multiple behavioral domains. For example, an intervention might address clinic workflow issues that waste time, while also providing communication skills training and question prompt lists.

The second generalizable lesson from this review is that methodological features of this communication literature make it challenging to compare studies or determine best practices. For example, these 88 studies utilized 188 different outcome measures, of which 156 were only utilized by individual studies. This great variability in outcome measures makes it difficult to compare results of studies, to interpret the clinical significance of small but statistically significant changes on non-validated measures, and ultimately to determine which studies are truly successful.

These challenges to interpretation are exacerbated by the variability in statistical methods employed by studies; most studies evaluated multiple outcome measures, some of which improved after the intervention. In some cases, every item on a measure or coding scheme was subjected to individual hypothesis testing, with the potential for numerous statistical comparisons within a single study. Alpha correction was seldom employed to account for multiple comparisons. While 11 studies in this review reported all null outcomes, only 18 studies reported all positive outcomes and the remaining 59 studies reported a mix of positive and null outcomes. Given the multiple comparisons, many of the positive findings could be merely results of chance, or could be erroneously positive because of flaws in the non-validated instruments that were employed. Furthermore, most outcomes were also assessed immediately after the intervention with only one comparison time point, thus calling to question the durability of these responses.

For some studies, it is reasonable to develop novel outcome measures, especially if the target of communication is not well represented in other areas of clinical communication (e.g. discussion of complementary and alternative medicine). However, some studies utilized novel measures where validated measures were available (e.g. quality of life, self-efficacy, decision-making preferences). Other methodological problems might be a result of limited resources and funding for such studies. For example, longitudinal follow up is essential to determining the sustainability of improvements in communication, but such follow up can require an infrastructure that exceeds the funding available for such studies.

However, our review has also highlighted several methodological strengths of this literature. First, these studies targeted a broad array of participants in the healthcare encounter, including nurses, doctors, patients, trainees, and combinations of all these. Furthermore, more than half of these interventions were evaluated in randomized controlled trials (RCTs), which provide a higher level of evidence for evaluating communication interventions. Also, greater than two-thirds of studies were multisite trials, which supports generalizability. However, no studies were reported from Eastern Europe, Latin America, South America, or Africa, highlighting a disparity in global communication research.

In terms of methodology and behavioral approach, a small number of exemplar studies stand out. The VOICE trial, for example, employed multimodal interventions including question prompt lists, physician communication training, and patient communication coaching in an RCT.[35] This multimodal intervention targeted 6 of 12 behavioral domains and 5 of 6 communication functions. Similarly, Paladino et al. published another exemplar study that employed multimodal interventions including question prompt lists and communication skills training in an RCT.[59] These interventions targeted 6 of 12 behavioral domains and 4 of 6 communication functions. Furthermore, this study repeated assessments every 2 months for 2 years or until the participant’s death. Despite the high quality of these studies, the results are underwhelming. The VOICE trial resulted in an improvement in a composite communication score that served as the primary outcome. However, this multimodal intervention did not lead to a difference in quality of life, clinicians’ responses to emotions, or provision of prognostic or treatment information. The study by Paladino et al. failed to improve the co-primary outcomes of goal-concordant care and peacefulness at the end of life, as well as the secondary outcomes of therapeutic alliance, depression, or survival. This study did find an improvement in patient-reported of anxiety.

We believe there are several ways to interpret these mostly negative results. First, targeting multiple domains might be ineffective in communication interventions. While this is a possibility, the failure of two studies certainly does not prove this point. Alternatively, characteristics of these studies might explain these negative results. These two studies employed rigorous methodologies and validated outcome measures. As such, these studies did not benefit from surrogate outcome measures or questionable statistical methodologies that might have provided positive outcomes, but little meaning. However, these studies might have failed simply because they did not target the right mix of behavioral levers. As we discussed earlier, workflow challenges might trump the best of intentions.

Future studies might also aim to incorporate advanced technology to facilitate communication. In this review, only 19% of studies incorporated any technology, and most of these uses were rudimentary (i.e. using a telephone to call a patient outside of the clinical encounter). The communication needs of patients can vary widely and may surpass the abilities of any single healthcare team member. As such, future studies should evaluate how some of these needs can be appropriately supported by technological interventions ranging from facilitative technologies (e.g. telemedicine or interactive patient portals) to stand-alone technologies (e.g. adaptive teaching modules or chatbots). For example, perhaps an interactive needs assessment identifies that a patient has many technical questions. But this patient also has concerns about which treatment will best fit his values and preferred lifestyle. If a computer interface can provide sufficient information and education to address the technical issues, then the patient will have more time discuss his values and preferences to appropriately support a shared decision. It is uncertain whether advancing technologies will help or hinder the clinician-patient relationship; this question should be answered with future studies.

Lastly, this scoping review only identified 3 studies that included pediatric clinicians, and no study specifically targeted pediatric or adolescent oncology. Children can vary widely in their cognitive and emotional development, which can affect their communication needs. Also, communication might serve different purposes for parents that are unique from their needs as patients.[28] Given these unique aspects of communicating with children and their parents, future work should aim to develop communication interventions specific to this population.

The results of this review should be considered in light of its limitations. First, we only included interventions that aimed to directly facilitate a communication interaction between a patient/parent and a clinician. As a result, many educational interventions and decision-aids were excluded from analysis. While these stand-alone interventions can be valuable, we were specifically interested in interventions that reinforced and supported the centrality of the clinical encounter. A second limitation was the potential overlap of behavioral domains and communication functions used in coding articles. To maximize consistency, we aimed to limit coding to the domains or functions most directly and explicitly targeted by the intervention without extrapolating to possible downstream effects of the intervention. For example, an educational seminar on communication skills with active practice sessions could potentially bolster “beliefs about capabilities” via “knowledge” and “skills”, however, we determined that availability of feedback for participants was an integral component of understanding one’s capabilities. Therefore, such a communication intervention would only be coded as targeting “beliefs about capabilities” if the workshop included feedback to participants. Similarly, if a training workshop included passive learning but no opportunity for active practicing of skills, we coded such interventions as targeting “knowledge” but not “skills.” These stringent criteria allowed for reproducible, consistent coding, but it is possible that more behavioral domains were engaged than we reported. Furthermore, we intentionally coded behavioral domains based on the description of the intervention provided in the manuscripts or supporting materials. In other words, we strove to avoid making assumptions about what domains an intervention was targeting when detail in the manuscript was insufficient. To illustrate, consider the domain “social/professional role and identity.” Some manuscripts provided sufficient details about the contents of the communication skills training sessions to facilitate coding of this domain. The communication skills workshop described by Liu, et al. in 2007,[118] for example, clearly targeted social/professional role and identity. In this intervention, they provided managerial support aimed at providing “nurses [with] positive feedback, establishing a peer-supportive atmosphere, implementing teaching rounds, building up role models, and conducting roleplaying within small groups in their workplace.” Many other interventions provided scant details about the content of their skills training sessions, and we suspect that few of these studies were as intentional about targeting the professional role and identity of clinicians. In the absence of compelling data from the manuscripts, we did not code communication skills training sessions as targeting this domain. Lastly, we excluded studies with fewer than 30 participants in the hopes of identifying studies that are more likely to have generalizable findings. However, some of these smaller studies might have had interesting findings to contribute.

In conclusion, changing communication behaviors is a challenging but essential goal in order to meet the needs of patients with serious illness. In this review, we have identified the need for further innovation in developing multimodal communication interventions that aim to engage multiple behavioral domains. In addition, we have identified methodological concerns with this body of communication intervention literature. In the future, we recommend that investigators view clinician-patient communication through the lens of behavioral change theories in order to develop interventions that can fulfill communication needs in feasible, specific, and sustainable ways.

## Supporting information

S1 Checklist PRISMA scoping review checklist. (PDF)
